# Effect of neoadjuvant chemotherapy combined with hyperthermic intraperitoneal perfusion chemotherapy on advanced gastric cancer

**DOI:** 10.3892/etm.2014.1599

**Published:** 2014-03-04

**Authors:** HAI-BIN CUI, HUAI-E GE, XI-YONG BAI, WEI ZHANG, YUAN-YUAN ZHANG, JUAN WANG, XING LI, LIAN-PING XING, SHENG-HU GUO, ZHI-YU WANG

**Affiliations:** 1 Department of Immunotherapy, Fourth Hospital of Hebei Medical University, Shijiazhuang, Hebei 050011, P.R. China; 2 Department of Oncology, Cangzhou Central Hospital, Cangzhou, Hebei 061001, P.R. China; 3 Department of Pathology, University of Rochester, New York, NY 14642, USA

**Keywords:** neoadjuvant chemotherapy, hyperthermic intraperitoneal perfusion, joint, gastric cancer

## Abstract

Neoadjuvant and hyperthermic intraperitoneal chemotherapies have been shown to be effective in the treatment of resectable advanced gastric cancer. The aim of the present study was to investigate the clinical efficiency and security of neoadjuvant chemotherapy in combination with hyperthermic intraperitoneal chemotherapy for the treatment of postoperative advanced gastric cancer. A total of 192 patients diagnosed with advanced gastric cancer were randomly divided into the following four groups (n=48 per group): Control, neoadjuvant chemotherapy, hyperthermic intraperitoneal perfusion chemotherapy and joint groups. The joint group received neoadjuvant chemotherapy combined with hyperthermic intraperitoneal perfusion chemotherapy. Complications, adverse reactions, recurrence rates within 2 years and the 1- and 3-year survival rates following surgery were observed. No significant differences were observed in the occurrence rates of I–II degree myelosuppression, III–IV degree myelosuppression, I–II degree nausea or III–IV degree nausea and vomiting among the four groups (P>0.05). The median progression-free survival times were 26, 31, 33 and 28 months in the control, neoadjuvant chemotherapy, hyperthermic intraperitoneal perfusion chemotherapy and joint groups, respectively (P<0.001). Compared with the control group, the recurrence-free 2-year survival rate of the joint group was significantly lower (P=0.04). The difference among the median survival times of the four groups was statistically significant (P=0.001). The 1-year survival rate of the joint group was significantly higher when compared with the control group and the difference was statistically significant (P=0.03). However, no statistically significant difference was identified among the 1-year survival rates of the four groups (P>0.05). Compared with the control group, the 3-year survival rates of the other three groups were significantly higher (P<0.05). Therefore, the results of the present study indicated that neoadjuvant chemotherapy combined with hyperthermic intraperitoneal perfusion chemotherapy for the treatment of advanced gastric cancer is well tolerated and exhibits improved compliance and efficiency.

## Introduction

Surgical resection is the only possible curative treatment for gastric cancer, however, this treatment is limited to stage I early gastric cancer cases. Neoadjuvant chemotherapy for the treatment of gastric cancer was initially reported by Wikle *et al* in 1989. Since then, numerous studies have demonstrated the safety and potency of this treatment, which functions to reduce the pathological stage and improve the surgical resection rate, particularly the R_0_ resection rate (1). In addition, neoadjuvant chemotherapy has been shown to reduce postoperative recurrence and metastasis, as well as improve the prognoses of patients with gastric cancer (1,2). Postoperative local recurrence and peritoneal metastasis of advanced gastric cancer are important factors that affect patient prognosis, with intraperitoneal metastasis being the most frequent outcome and cause of mortality in advanced gastric cancer. Studies have shown that for advanced gastric cancer with penetrated serosa, neoadjuvant chemotherapy reduces intraperitoneal recurrence and metastasis, thus, increases the overall survival rate of patients (3). In addition, hyperthermic intraperitoneal perfusion chemotherapy has been shown to effectively eliminate cancer cells that escape to the peritoneal cavity, thus, preventing peritoneal local recurrence and metastasis (4). In the present study, preoperative neoadjuvant chemotherapy was combined with postoperative hyperthermic intraperitoneal perfusion chemotherapy for the treatment of advanced gastric cancer.

## Materials and methods

### Ethics

The study was approved by the Medical Ethics Committee of the Cangzhou Central Hospital (Cangzhou, China) and informed consent was provided by all patients.

### Clinical data and grouping

A total of 192 patients that had been diagnosed with advanced gastric cancer and that had undergone surgery between January 2006 and January 2010 at the Department of Oncological Surgery, Cangzhou Central Hospital, were enrolled in the study. Inclusion criteria firstly included a confirmed diagnosis of gastric cancer by gastroscopy biopsy and histopathological examinations, with the Tumor, Node and Metastasis classification identifying the tumors as IIIA or IIIB, according to the American Joint Committee on Cancer staging system (5), without the presence of hepatic, pulmonary, cerebral or bone metastasis. Secondly, the tumors were evaluated to be stage IIIA or IIIB by endoscopic ultrasound (EUS) and computed tomography (CT) scans that revealed at least one measurable lesion. Thirdly, patients were required to be aged between 18 and 75 years and have a Karnofsky Performance Status score of ≥60. Finally, patients had not undergone chemotherapy prior to enrollment and no surgical or chemotherapeutic contradictions were present in the preoperative examinations. Patients were excluded from the study if they had residual gastric cancer or had undergone a laparotomy. The patients were randomly divided into four groups (Tables I and II). The control group comprised 48 cases (males, 21; females, 27; age, 39–72 years; average age, 56 years) of which 28 cases were classified as stage IIIA and 20 cases were classified as stage IIIB. In addition, 16 cases had highly or moderately differentiated adenocarcinomas, 25 cases had poorly or undifferentiated adenocarcinomas and 7 cases had mucinous adenocarcinoma or mucinous cell carcinoma. Surgical treatment without postoperative chemotherapy was performed in this group. The neoadjuvant chemotherapy group comprised 48 cases (males, 19; females, 29; age, 41–69 years; average age, 55 years) of which 29 cases were classified as stage IIIA and 19 cases were classified as stage IIIB. In addition, 15 cases had highly or moderately differentiated adenocarcinomas, 21 cases had poorly or undifferentiated adenocarcinomas and 12 cases had mucinous adenocarcinoma or mucinous cell carcinoma. Preoperative neoadjuvant chemotherapy and surgical treatment were performed in this group, but without postoperative hyperthermic intraperitoneal perfusion chemotherapy. The hyperthermic intraperitoneal perfusion chemotherapy group comprised 48 cases (males, 22; females, 26; age, 39–70 years; average age, 53 years) of which 25 cases were classified as stage IIIA and 23 cases were classified as stage IIIB. In addition, 14 cases had highly or moderately differentiated adenocarcinomas, 22 cases had poorly or undifferentiated adenocarcinomas and 12 cases had mucinous adenocarcinoma or mucinous cell carcinoma. Surgical treatment and postoperative hyperthermic intraperitoneal perfusion chemotherapy were performed in this group, but without neoadjuvant chemotherapy. The joint group comprised 48 cases (males, 20; females, 28; age, 42–68 years; average age, 55 years) of which 27 cases were classified as stage IIIA and 21 cases were classified as stage IIIB. In addition, 18 cases had highly or moderately differentiated adenocarcinomas, 21 cases had poorly or undifferentiated adenocarcinomas and 9 cases had mucinous adenocarcinoma or mucinous cell carcinoma. Preoperative neoadjuvant chemotherapy, surgical treatment and hyperthermic intraperitoneal perfusion chemotherapy were performed in this group. Differences among the clinical data of the four groups exhibited no statistical significance (P>0.05), but had comparability (Tables I and II).

### Therapeutic project

Neoadjuvant chemotherapy was performed with 135 mg/m^2^ paclitaxel administered via an intravenous drip on day 1, 20 mg/m^2^ cisplatin administered via an intravenous drip between day 1 and 5 and 0.8–1.0 g tegafur administered per day via an intravenous drip between day 1 and 5. The course was repeated every 3 weeks. In the neoadjuvant chemotherapy group, the efficacy was evaluated by clinical conditions, EUS and CT scans every 2 weeks, according to the response evaluation criteria in solid tumors (6). If the evaluation determined the treatment to be effective or stable, the regimen continued to the end of the fourth course, 3 weeks after which surgery was performed. Furthermore, following surgery, an additional three courses of treatment with the original regimen were administered. By contrast, if tumor progression occurred, immediate surgical treatment was performed. This was followed by four courses of a different regimen called ‘ECF’, which contained 50 mg/m^2^ epirubicin and 60 mg/m^2^ cisplatin administered via an intravenous drip on day 1 and 600 mg/m^2^ fluorouracil administered via an intravenous drip between day 1 and 3. Postoperative hyperthermic intraperitoneal perfusion chemotherapy started on day 1 or 2 following surgery, according to the recovery status of the patient following the radical resection of the gastric cancer. This chemotherapy was performed for 90 min per day for four consecutive days. In the neoadjuvant chemotherapy and joint groups, the surgical treatment usually started 3 weeks following the last course of neoadjuvant chemotherapy. In the control and neoadjuvant chemotherapy groups, systemic chemotherapy was performed between week 2 and 3 following surgery. While in the hyperthermic intraperitoneal perfusion chemotherapy and joint groups, chemotherapy did not start until 1 month following surgery.

### Intraoperative placement of hyperthermic perfusion catheters

During the radical resection of gastric cancer, silicone hyperthermic perfusion catheters (Jilin Morestep Medical Device Co., Ltd., Changchun, China) were placed at the bilateral paracolic sulcus, diaphragmatic surface of the liver and the splenic recess separately. The catheters had side pores on the front end, and inside and outside diameters of 8 and 10 mm, respectively. An additional drainage catheter was placed at the upper abdomen and exited at the paraumbilical region.

### Hyperthermic perfusion chemotherapy

Chemotherapy was conducted using an RHL-2000B Chemo-hyperthermia perfusion system (Jilin Morestep Medical Device Co., Ltd.). Treatment started on day 1 or 2 following surgery, according to the recovery status of the patient. Chemotherapy was performed for 90 min per day for four consecutive days. On day 1 and 4, the intraperitoneal hyperthermic perfusate consisted of 60 mg/m^2^ cisplatin and 3,000 ml normal saline, while on day 2 and 3, the perfusate consisted of 0.75 g fluorouracil and 3,000 ml normal saline. In addition, 10 mg dexamethasone and 10 ml lidocaine (2%) were routinely added to the perfusate in order to reduce peritoneal reactions. The perfusion machine, circulation pump and heater were powered at 38°C, which was reached prior to therapy. Next, the circulatory pipes were connected to the abdominal drainage catheters via a two-in-three-out manner, through which the hyperthermic perfusate was infused into the abdominal cavity. The temperature of the perfusate was then elevated to and stabilized at 41–43°C using a temperature control system that lasted for 90 min. During hyperthermic perfusion, the patients underwent electrocardiogram monitoring to facilitate real-time adjustment of the perfusion machine according to the situation. The patient also routinely received intramuscular injections of dolantin and diazepam to improve tolerance. Following therapy, ~1,000 ml hyperthermic perfusate was left in the abdominal cavity and the remaining perfusate was drained.

### Evaluation of efficiency and adverse reactions

Alimentary tract reactions and marrow function were evaluated according to the World Health Organization’s standard toxicity assessment. In addition, regular re-examinations, including gastroscopy, chest and abdominal CT scans and tumor biomarkers, were conducted to obtain information on intraperitoneal recurrence within 2 years and the 1- or 3-year survival rates.

### Statistical analysis

SPSS 18.0 software (SPSS, Inc., Chicago, IL, USA) was used to conduct statistical analysis of the experimental data. Quantitative data were analyzed using the χ^2^ and Fisher’s exact tests. Progression-free survival and overall survival rates were calculated using the Kaplan-Meier method. Logrank tests were used to analyze the significance of difference among the progression-free and overall survival rates of the four groups, while the t-test was employed for the comparison of the 2-year progression-free survival and 1- and 3-year survival rates of the control group. P<0.05 was considered to indicate a statistically significant difference.

## Results

### Adverse reactions and complications

No patient succumbed during surgery. There was not a marked difference between the occurrence rates of I–II degree myelosuppression, III–IV degree myelosuppression, I–II degree nausea and III–IV degree nausea and vomiting among the four groups (P>0.05; Table III).

### Comparison of efficiency in each group

**Figure 1:**
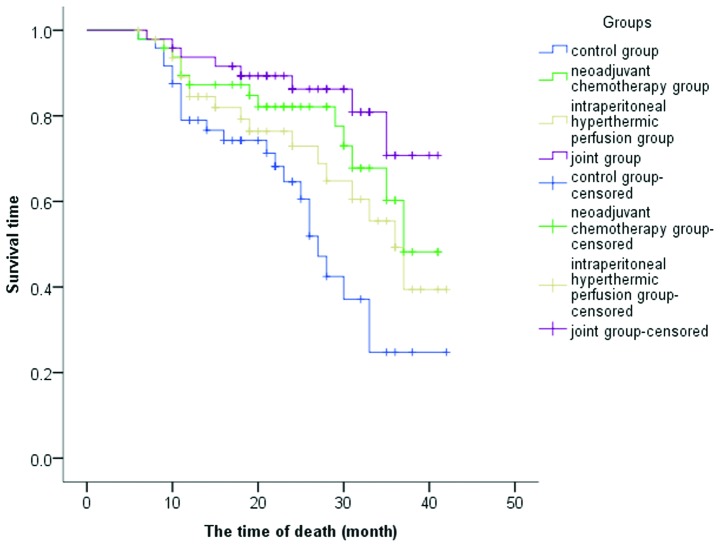
Non-recurrence survival rate and duration following surgery of the patients in the four groups. Median progression-free survival times were 26, 28, 31 and 33 months in the control, neoadjuvant chemotherapy, hyperthermic intraperitoneal perfusion chemotherapy and joint groups, respectively; the difference was statistically significant (χ^2^, 14.63; P<0.001).

There were 1, 2, 1 and 0 cases lost during the follow-ups in the control, neoadjuvant chemotherapy, hyperthermic intraperitoneal perfusion chemotherapy and joint groups, respectively. The median progression-free survival times were 26, 28, 31 and 33 months in the control, neoadjuvant chemotherapy, hyperthermic intraperitoneal perfusion chemotherapy and joint groups, respectively; the difference was statistically significant (χ^2^, 14.63; P<0.001; Fig. 1).

There were 16, 11, 8 and 6 cases that exhibited recurrence within 2 years in the control, neoadjuvant chemotherapy, hyperthermic intraperitoneal perfusion chemotherapy and joint groups, respectively. Thus, the recurrence-free 2-year survival rates in the corresponding groups were 66.67, 77.08, 83.33 and 87.5%, respectively. Compared with the control group, the recurrence-free 2-year survival rate of the joint group was significantly lower (P=0.04; Table IV).

### Comparison of the mortality rates in each group

**Figure 2:**
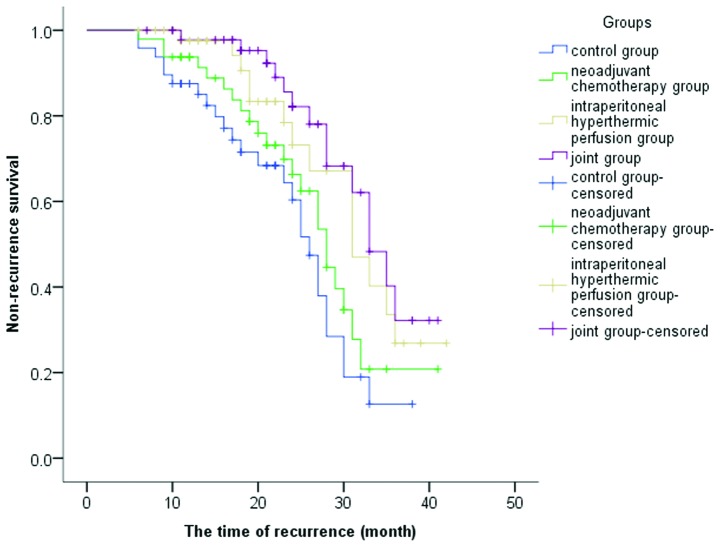
Survival time curves of the patients in each group. Median survival times were 27, 33, 32 and 36 months in the control, neoadjuvant chemotherapy, hyperthermic intraperitoneal perfusion chemotherapy and joint groups, respectively; the differences between the four groups were statistically significant (χ^2^, 10.37; P=0.001).

Median survival times were 27, 33, 32 and 36 months in the control, neoadjuvant chemotherapy, hyperthermic intraperitoneal perfusion chemotherapy and joint groups, respectively; the differences among the four groups were statistically significant (χ^2^, 10.37; P=0.001; Fig. 2). The 1- and 3-year survival rates were 79 and 25% in the control group, 87.3 and 60.3% in the neoadjuvant chemotherapy group, 84.5 and 49.3% in the hyperthermic intraperitoneal perfusion chemotherapy group and 93.7 and 70.8% in the joint group, respectively. The 1-year survival rate of the joint group was significantly higher when compared with the control group and the difference was statistically significant (P=0.03). However, no statistically significant differences were observed among the 1-year survival rates of the neoadjuvant chemotherapy, hyperthermic intraperitoneal perfusion chemotherapy and control groups (P>0.05; Table IV). However, when compared with the control group, the 3-year survival rates of the three treatment groups were significantly higher (P=0.002).

## Discussion

Clinically, advanced gastric cancer is most commonly observed in Chinese patients (7). Due to the anatomical features of the stomach, advanced gastric cancer is prone to local recurrence and distal metastasis. Furthermore, when the tumor penetrates the serosa and results in peritoneal implantation, the recurrence and metastasis rates are markedly elevated, rendering a considerably lower 5-year survival rate. Therefore, a favorable outcome is difficult to achieve through a surgical approach alone. The emergence of neoadjuvant chemotherapy has improved the prospects of patients that were previously inoperable since the chemotherapy shrinks the tumor and lowers the clinical staging, which is beneficial for the resection of local lesions. In addition, theoretically, neoadjuvant chemotherapy can pretreat the possible micro-metastasis preoperatively. Hyperthermic intraperitoneal perfusion chemotherapy can effectively eliminate cancer cells that have escaped to the peritoneal cavity, thus, preventing peritoneal local recurrence and metastasis. In previous years, numerous studies have shown that neoadjuvant and hyperthermic intraperitoneal perfusion chemotherapies can delay the recurrence and metastasis of gastric cancer without increasing adverse reactions, thus, improving the median progression-free survival times and overall survival rates, (4,8–10).

In the present study, no patients succumbed during surgery in any of the four groups. The myelosuppression and alimentary tract adverse reactions were predominantly grade I–II and were successfully treated without affecting the courses of chemotherapy. No statistically significant differences were observed in the occurrence of adverse reactions and complications that were controlled with proper medication. In the follow-up, statistically significant differences were observed among the progression-free survival rates of the four groups. Compared with the control group, the 2-year progression-free survival rate of the joint group was significantly lower. According to the UK NCRI MAGIC trial, 503 patients with resectable gastric cancer were randomly divided into a preoperative chemotherapy + surgery + postoperative chemotherapy group and a surgery group. The results revealed that the total response and 5-year survival rates of the former group were significantly different when compared with the control group (11). In the French FFCD trial, 224 operable patients were divided into a perioperative 5-fluorouracil plus cisplatin chemotherapy group (113 cases) and a surgery group. The results revealed there to be significant differences in the 5-year disease-free survival and overall survival rates between the two groups (12). Therefore, the two previous studies indicate that neoadjuvant chemotherapy can ameliorate patient prognosis. In the present study, paclitaxel, cisplatin and tegafur were combined to perform neoadjuvant chemotherapy for advanced gastric cancer. There was no statistically significant difference in preoperative staging for each patient enrolled in this trial and every patient underwent the whole course of chemotherapy. The results were similar to those of the MAGIC and French FFCD trials and demonstrated that in the neoadjuvant chemotherapy and joint groups, the median survival times were improved and the local recurrence rates were reduced.

Contemporary hyperthermic intraperitoneal perfusion chemotherapy is usually performed once during surgery, thus, the effectiveness of the procedure may be impaired by the limited treatment time. In the current study, four to five drainage catheters were embedded intraoperatively, making the perfusion procedure more fluent and convenient. Following surgery, early hyperthermic intraperitoneal perfusion chemotherapy was performed once a day for 4 days. This regimen was selected as, theoretically, more tumor cells were able to be drained out of the peritoneal cavity, enhancing the efficacy of the intraperitoneal chemotherapeutic drugs (13).

The mechanism behind recurrence in advanced gastric cancer requires further elucidation. It is generally hypothesized that the phenomenon is associated with the following aspects (14). Firstly, intraperitoneal free cancer cells are important initiators of recurrence in the peritoneal cavity. Secondly, intraoperative injury of the surface of intraperitoneal organs facilitates the implantation and spread of intraperitoneal free cancer cells. Finally, the stress of anesthesia and surgery results in the immunity of the patient being suppressed following surgery, rendering the escape of intraperitoneal free cancer cells from the surveillance of the immune system. The mechanisms underlying the prevention effects of hyperthermic intraperitoneal perfusion chemotherapy on postoperative recurrence and metastasis in the peritoneal cavity are as follows (15). Firstly, a large amount of circulatory perfusate dilutes the concentration of intraperitoneal free cancer cells and rinses the free cancer cells out of the peritoneal cavity. Secondly, hyperthermia exhibits a synergic effect with chemotherapy, which is possibly a result of hyperthermia enhancing the transportation of tumor cells and the absorbance of platinum-based chemotherapeutic drugs. Thus, the cancer cells are sensitized to chemotherapy. Thirdly, hyperthermia causes the denaturation of surface proteins on cancer cells, altering the permeability of the cytomembrane and enhancing the absorbance of drugs and their anticancer effect (16). Finally, the hyperthermic perfusate is absorbed by the peritoneum and is transported to the liver via the portal vein. This eliminates the cancer cells located within the portal vein and prevents hepatic metastasis. A meta-analysis revealed that hyperthermic intraperitoneal perfusion chemotherapy improves the overall survival rate of patients, but increases the risk of complications, including myelosuppression (17). However, in the present study, no statistically significant differences were observed in the occurrence of adverse reactions among the four groups. Compared with the control group, the 3-year survival rates of the joint and hyperthermic intraperitoneal perfusion chemotherapy groups were significantly higher (P<0.05). Therefore, these groups had an improved prognosis, which is in accordance with the results of a previous study by Wang *et al* (9). In addition, Votanopoulos *et al* found that hyperthermic intraperitoneal perfusion chemotherapy improved the survival rate of elderly patients (18).

In conclusion, the present study demonstrated that the occurrence rate of adverse reactions and complications in the joint group was not statistically different from those of the other three groups. The follow-up study indicated that the joint group exhibited a significantly lower 2-year postoperative recurrence rate and a higher overall survival rate. Therefore, neoadjuvant chemotherapy combined with hyperthermic intraperitoneal perfusion chemotherapy improves progression-free survival and overall survival rates, and is a safe, effective and feasible treatment for resectable advanced gastric cancer. However, the number of cases enrolled in the present study was small. Although the time span was large, the follow-up time was only 3 years, which may have resulted in the efficacy of the combined therapy not being fully exhibited. In addition, the 5-year survival rate remains uninvestigated. Therefore, further follow-up studies are required. In conclusion, neoadjuvant chemotherapy combined with hyperthermic intraperitoneal perfusion chemotherapy for the treatment of advanced gastric cancer has been shown to be well tolerated with improved compliance and efficiency.

## Figures and Tables

**Table I:** Comparison of gastric cancer pathology types in each group.

Groups	Moderately/well differentiated adenocarcinoma	Poorly/undifferentiated adenocarcinoma	Mucinous adenocarcinoma or mucinous cell carcinoma	χ^2^	P-value
Control	16	25	7		
Neoadjuvant chemotherapy	15	21	12		
Hyperthermic intraperitoneal perfusion chemotherapy	14	22	12		
Joint	18	21	9	2.84	0.83

**Table II:** Gastric cancer stages of each group.

Groups	Stage IIIA	Stage IIIB	χ^2^	P-value
Control	28	20		
Neoadjuvant chemotherapy	29	19		
Hyperthermic intraperitoneal perfusion chemotherapy	25	23		
Joint	27	21	0.74	0.86

**Table III:** Comparison of adverse events in each group.

Parameter	Grade I–II myelosuppression	Grade III–IV myelosuppression	Grade I–II nausea and vomiting	Grade III–IV nausea and vomiting
Control	25	1	18	0
Neoadjuvant chemotherapy	27	2	21	1
Hyperthermic intraperitoneal perfusion chemotherapy	26	1	23	1
Joint	30	3	25	2
χ^2^	1.19		2.25	
P-value	0.76	0.84	0.52	0.9

**Table IV:** Comparison of recurrence and survival rates in each group.

Groups	Recurrence rate in 2 years, %	1-year survival rate, %	3-year survival rate, %
Control	33.33 (16/48)	79.16 (38/48)	35.41 (17/48)
Neoadjuvant chemotherapy	22.92 (11/48)	87.50 (42/48)	62.50 (30/48)
Hyperthermic intraperitoneal perfusion chemotherapy	16.67 (8/48)	85.41 (41/48)	58.33 (28/48)^a^
Joint	12.50 (6/48)^a^	93.75 (45/48)^a^	75.00 (36/48)^b^

a P<0.05, vs. control;

b P<0.01, vs. control.
